# Possible Threats of IgA Vasculitis in Children: One Center Experience

**DOI:** 10.15388/Amed.2024.31.2.4

**Published:** 2024-12-04

**Authors:** Eglė Lanzbergaitė-Manuilova, Skirmantė Rusonienė, Augustina Jankauskienė

**Affiliations:** Pediatric Center, Institute of Clinical Medicine, Faculty of Medicine, Vilnius University, Vilnius, Lithuania

**Keywords:** IgA vasculitis, gastrointestinal involvement, kidney involvement, laboratory predictors, Raktažodžiai: IgA vaskulitas, virškinamojo trakto pažeidimas, inkstų pažeidimas, laboratoriniai prognostiniai rodikliai

## Abstract

**Introduction:**

Immunoglobulin A vasculitis (IgAV) is the most common vasculitis in children. Although typically self-limiting, IgAV may result in serious complications. Our objective was to evaluate the incidence, clinical features, laboratory predictors and outcomes of IgA vasculitis with gastrointestinal (GI) and kidney involvement.

**Methods:**

Medical records of patients <18 years of age with newly diagnosed IgAV between 2013 and 2021 in a single center were analyzed. Demographic, clinical, laboratory data, and incidence of GI and kidney involvement data were analyzed. As laboratory predictors, neutrophil, lymphocyte, platelets count, mean platelet volume (MPV) and neutrophil-to-lymphocyte ratio (NLR), platelet-to-lymphocyte ratio (PLR) were calculated.

**Results:**

240 patients with IgAV were included. GI involvement was in 104 patients (43.3%), whereas kidney involvment in 21 patients (8.8%). Age was the only variable associated with increased odds of kidney involvement (OR 3.5, 95% confidence interval 1.39–8.56, p=0.009). None of the laboratory predictors or other tested variables was associated with kidney involvement in univariable logistic regression. The neutrophil and lymphocyte count, NLR and PLR levels were found to be significantly higher in children with GI involvement. There were no bad outcomes: lethal outcome or chronic kidney disease for the patients with GI and kidney involvement in recent study. During two years of surveillance after IgAV diagnosis, 11 cases (4.6%) had indications for kidney biopsy and were diagnosed with IgAV nephritis.

**Conclusions:**

Older children were more likely to have kidney disease. Easy obtained laboratory parameters such as NLP, PLR could help to predict GI involvement in early disease stage, but had no value for predicting kidney involvement.

## Abstract (it)

**Įvadas:**

Imunoglobulino A vaskulitas (IgAV) yra dažniausias vaskulitas vaikų amžiuje. Nors IgAV paprastai praeina savaime, jis gali sukelti ir rimtų komplikacijų. Mūsų tyrimo tikslas įvertinti vaikų, sergančių IgAV ir turinčių virškinamojo trakto (GI) ir (ar) inkstų pažeidimą, dažnumą, klinikinius požymius, galimus prog-nostinius laboratorinius rodiklius ir ligos baigtis.

**Metodai:**

Tyrimo metu analizuojami jaunesnių nei 18 metų pacientų, kuriems 2013–2021 m. buvo naujai diagnozuotas IgAV, vieno centro duomenys. Analizuoti demografiniai, klinikiniai, laboratoriniai rodikliai, GI ir inkstų pažeidimo dažnis. Kaip galimi prognostiniai laboratoriniai rodikliai buvo apskaičiuotas neutrofilų, limfocitų, trombocitų skaičius, vidutinis trombocitų tūris (MPV), neutrofilų ir limfocitų santykis (NLR), trombocitų ir limfocitų santykis (PLR).

**Rezultatai:**

įtraukta 240 pacientų, sergančių IgAV. Virškinamojo trakto pažeidimas buvo nustatytas 104 pacientams (43,3 %), o inkstų pažeidimas – 21 pacientui (8,8 %). Amžius buvo vienintelis kintamasis, susijęs su padidėjusia inkstų pažeidimo tikimybe (šansų santykis 3,5, 95 %, pasikliautinis intervalas 1,39–8,56, p = 0,009). Nė vienas iš laboratorinių rodiklių ar kitų patikrintų kintamųjų nebuvo susijęs su inkstų pažeidimu vienanarėje logistinėje regresijoje. Nustatyta, kad neutrofilų ir limfocitų skaičius bei NLR ir PLR santykis yra statistiškai reikšmingai didesni vaikams, turintiems GI pažeidimą. Šiame tyrime nebuvo jokių blogų baigčių: mirtina baigtis arba lėtinė inkstų liga pacientams, sergantiems IgAV su GI ar inkstų pažeidimu. Per dvejus metus po IgAV diagnozės nustatymo 11 pacientų (4,6 proc.) pagal indikacijas atlikta inkstų biopsija ir diagnozuotas IgAV nefritas.

**Išvados:**

vyresni vaikai dažniau turėjo inkstų pažeidimą nei jaunesni. Lengvai gaunami laboratoriniai parametrai, tokie kaip NLP, PLR, galėtų padėti numatyti GI pažeidimą ankstyvoje ligos stadijoje, tačiau neturėjo reikšmės prognozuojant inkstų pažeidimą.

## Introduction

Immunoglobulin A vasculitis (IgAV), known as Henoch–Schonlein purpura, is the most common vasculitis in children with an annual incidence rate of 3–26.7/100.000, occurring at any age [1]. The estimated incidence rates among children are 2 to 33 times greater than those in adults [2], 75%–90% of pediatric patients are below 10 years old, and the most frequent being 4–7 years of age, with an incidence up to 70.3/100.000 [3]. Certain children age is at most particular risk for pathogenic infections.

IgAV is most often characterized by a mild, self-limiting course and good prognosis, but in some cases, serious complications such as severe gastrointestinal (GI) involvement – GI bleeding, bowel edema, intussusception, and intestinal perforation or kidney involvement – may increase the morbidity and mortality in children with IgAV [1-2, 4-6]. The short-term prognosis of the disease depends on the severity of a potential acute involvement of the gastrointestinal tract, but long-term prognosis is dependent on the extent of kidney damage. Up to 50% of pediatric patients develop nephritis within 4 to 6 weeks of initial presentation. The risk of progression to end-stage kidney disease is reported from 4% after 4.6 years in Europe [7] to 11%–14% after 15 years of follow-up in Asia countries [8-9].

Considering the possibility of serious complications there is need to identify patients at a higher risk of developing severe extracutaneous symptoms. Not expensive and easily available laboratory parameters that can be used to assess the severity of systemic inflammation include the following ratios: neutrophil-to-lymphocyte (NLR) and platelet-to-lymphocyte (PLR). NLR is an inflammatory marker, used to assess systemic inflammation in various diseases. Few studies indicate significant differences in NLR and PLR values in IgAV patients compared to healthy subjects. Elevated NLR and PLR, as well as low mean platelet volume (MPV), appear to be indicators related to GI bleeding or kidney manifestations in the course of IgAV [3].

The aim of this study is to analyze the general and clinical characteristics of IgAV patients, evaluate their hematological inflammatory markers in predicting GI and kidney involvement and long-term outcomes of IgAV patients.

## Methods

Data was collected at a tertiary hospital inpatient department in the period from January 2013 to December 2021. Inclusion criteria were all children below 18 years of age with confirmed IgAV diagnosed according by EULAR/PRINTO/PRES criteria [10]. Patients were excluded if they had hematological disorders, coexisting systemic connective tissue disease, malignancy, any chronic kidney or GI diseases. New occurrence of IgAV was evaluated annually and compared between pre-COVID-19 period (2013 to 2020 February) and the COVID-19 period (March 2020 to December 2021). Demographics, seasonal distribution, clinical, main laboratory data, incidence of gastrointestinal, kidney involvement were analyzed. Outcome of kidney disease were assessed during 2 years of follow up.

Patients with purpura were divided into groups according to the presence of GI in one group and kidney involvement in the other. GI involvement was defined as the presence of abdominal pain or occult blood in stool, melena. Kidney involvement or nephritis was defined as the presence of hematuria (hematuria of >5 erythrocytes/high-power field) and/or proteinuria (less than 30 mg/mmol on a spot morning sample, which considered insignificant) and/or impaired kidney function.

To investigate the differences in clinical manifestation according to the age, patients were categorized in two groups: younger than or 7 years old and older than 7 years.

Clinical tests were done according the hospital protocol: blood, urine and stool samples were collected at the time of the patient’s admission to the hospital. Neutrophil, lymphocyte, platelets count, MPV was obtained from the complete blood count test results. NLR was calculated by dividing the neutrophil count by the lymphocyte count, and PLR was calculated by dividing the platelet count by the lymphocyte count. In two years after disease presentation, all patients, who showed for return visit due to remaining or new additional symptoms, was monitored.

152 healthy children matching by age without acute infectious diseases or without any chronic disease, but to whom blood test was performed (before planned surgery or prophylactic testing patients), randomly selected, served as a control group comparing laboratory tests.

Written informed consent was obtained from patients’ parents and the study protocol was approved by Vilnius Regional Bioethics Committee (SHN-IgAN-2018).

## Statistical analyses

All data were analyzed using the SPSS v. 21 software package (IBM). Mean ± standard deviation (SD) was used to describe continuous data. To compare continuous data between two groups Mann–Whitney U test was applied to calculate intergroup differences. Pearson chi-square test was used for categorical variables. The two sample Kolmogorov–Smirnov test is a nonparametric test that compares the cumulative distributions of two data sets

Logistic regression analysis was performed for multivariate analysis. A receiver operating characteristic (ROC) curve was performed to examine the prognostic utility of NLR, PLR and MPV and to identify the optimal cut-off value. Statistical analysis was performed using the Kruskal–Wallis nonparametric analysis of variance test. Analysis of variance (ANOVA) and Kruskal–Wallis test were used to compare results of laboratory parameters among groups, and prediction models were built by using logistic regression analysis. Results with p < 0.05 were considered statistically significant.

## Results

**Figure 1:**
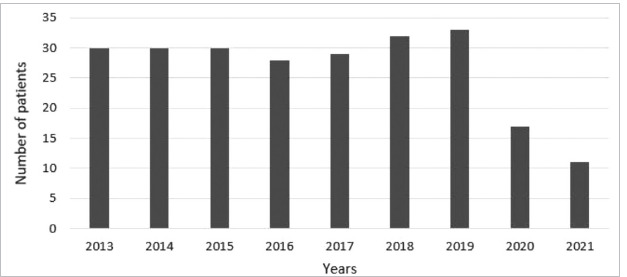
Number of newly diagnosed IgAV patients in years

Two hundred forty children were diagnosed with IgAV in the period of January 2013 to December 2021. The annual distribution of patients is shown in Fig. 1. The annual mean number of patients decreased by 53% during COVID-19 pandemic years (30 patients/year vs. 14 patients/pandemic year). It could have happened because of lower exposure to infectious agents due to lockdown or lower diagnosis rate because of difficulties to contact with healthcare providers.

**Figure 2:**
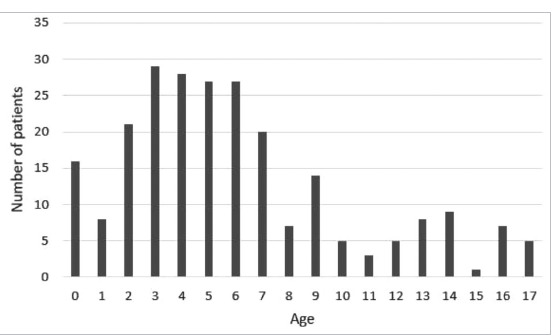
Age distribution of IgAV patients

The main clinical characteristics of IgAV patients are summarized in Table 1. The IgAV occurred during all seasons, but winter and spring were the high seasons. Upper respiratory tract infections were the most common trigger of the disease (51%), though about 40% of cases of triggers remain unknown. Age distribution is presented with relatively high incidence of patients aged 2 to 7 years (73.3%) (Fig. 2).

**Table 1:** Clinical features of IgAV patients (N =240)

Characteristic	Value number (%)
Age, mean ± SD	6.05 ± 4.28
Gender, male/female	135/105 (1.29/1)
Clinical manifestation	
purpura	240 (100.0)
GI involvement	104 (43.3)
kidney involvement	21 (8.8)
only purpura	115 (47.9)
Seasons:	
winter	73 (30.4)
spring	77 (32.1)
summer	23 (9.6)
autumn	67 (27.9)
Etiology:	
URI	107 (44.6)
Streptococcal infection	9 (3.8)
astrointestinal infections	17 (7.1)
Insect bite	1 (0.4)
COVID-19 infection	4 (1.7)
influenza	3 (1.3)
unknown	95 (39.6)

Abbreviations: IgAV, immunoglobulin A vasculitis; N, number of patients; URI, upper respiratory infection, SD, standard deviation

All patients diagnosed with IgAV had purpura, but severe purpura occurred in 10% of cases, while there were cases of atypical localizations such as face, scrotum, underarms, and in one case the whole body.

Immunoglobulin A vasculitis with nephritis (IgAVN) at the time of presentation was identified in 21 patients (8.8%) and included isolated hematuria (7.1%) or proteinuria with hematuria (1.7%). All patients with kidney involvement had normal kidney function. Patients with IgAVN were older (8.6±1.1 vs 5.8±0.3 years, p=0.021) with male predominance (male-to-female ratio 2.5:1). During two years of surveillance after IgAV diagnosis, 11 cases (4.6%), had indications for kidney biopsy and were diagnosed with IgAV nephropathy. These patients were not necessary the same as patients with IgAVN involvement at disease presentation.

The neutrophil, lymphocyte, platelet counts and NLR and PLR values were significantly higher in the IgAV group compared with control group (Table 2). There was no significant statistical difference in the value of MPV between the patient and the control groups.

**Table 2:** Comparison of laboratory parameters between children with IgAV and control group.

Variable	IgAV group, N=240	Control group, N= 152	P Value
Age, y	6.05 ± 4.28	11.27 ± 4.46	0.144
Gender, male/female	134/106 (1.26:1)	90/62 (1.45:1)	0.531
Neutrophil count	5.77 (0.64; 23.68)	3.21(1.14; 13.38)	0.001
Lymphocyte count	3.37 (0.44; 14.53)	2.54 (0.99; 10.91)	0.010
Platelet count	339.00 (129.0; 912.0)	276.00 (117.0; 587.0)	0.000
NLR	1.7 (0.08; 22.36)	1.21 (0.19; 6.16)	0.000
MPV	9.4 (7.5; 96.0)	9.8 (7.7; 12.1)	0.533
PLR	111.4 (15.2; 440.0)	107.5 (29.3; 334.3)	0.013

Abbreviations: IgAV, immunoglobulin A vasculitis; NLR, neutrophil-to-lymphocyte ratio; MVP, mean platelet volume; PLR, platelet-to-lymphocyte ratio. Values are presented as median and range.

Age was the only variable associated with increased odds of kidney involvement (OR 3.5, 95% CI 1.39–8.56, p=0.009). None of the tested variables (neutrophil, lymphocyte and platelet count, NLR, PLR, gender, seasonality, gastrointestinal involvement) were associated with kidney involvement in univariable logistic regression (all p<0.05).

The neutrophil and lymphocyte count, NLR and PLR levels were found to be significantly higher in children with GI involvement compared to those without GI involvement. There was no difference in platelet count and MPV (Table 3).

**Table 3:** Nonparametric statistical analysis for IgAV children combined with GI and IgAVN

	Gastrointestinal involvement			IgAVN		
	Yes, N = 104	No, N = 136	P Value	Yes, N=21	No, N=219	P Value
Age, y	5.96 ± 3.87	6.13 ± 4.58	0.770	8.57±5.18	5.81±4.12	0.005
Gender, male/female	59/45	76/60	0,500	11/10	124/95	0.440
Neutrophil count	6.75 (1.34; 23.68)	5.14 (0.64; 18.5)	0.004	6.87 (3.97; 23.68)	5.71 (0.64; 22.12)	0.065
Lymphocyte count	3.12 (0.44; 14.53)	3.57 (0.72; 11.60)	0.032	3.35 (0.96; 6.35)	3.37 (0.44; 14.53)	0.710
Platelet count	351.5 (129.0; 912.0)	320.0 (142.0; 764.0)	0.060	299.0 (166.0; 680.0)	341.00 (129.0; 912.0)	0.219
NLR	1.89 (0.14; 22.36)	1.46 (0.08; 7.99)	0.002	1.91 (1.07; 8.36)	1.60 (0.08; 22.36)	0.062
MPV	9.5 (7.5; 12.0)	9.3 (7.8; 10.7)	0.571	9.4 (7.5; 11.4)	9.40 (7.8; 10.7)	0.828
PLR	109.02 (15.2; 440.0)	92.71 (21.8; 432.4)	0.002	92.9 (44.1; 266.7)	100.00 (15.2; 440.0)	0.754

Abbreviations: GI, gastrointestinal involvement; IgAV, immunoglobulin A vasculitis; IgAVN, immunoglobulin A vasculitis with nephritis; N, number of patients; NLR, neutrophil-to-lymphocyte ratio; MVP, mean platelet volume; PLR, platelet-to-lymphocyte ratio. Values are presented as median and range.

The blood parameters – neutrophil, lymphocyte and platelet count, NLR, MPV and PLR – were similar between children with and without renal involvement (Table 3).

**Figure 3:**
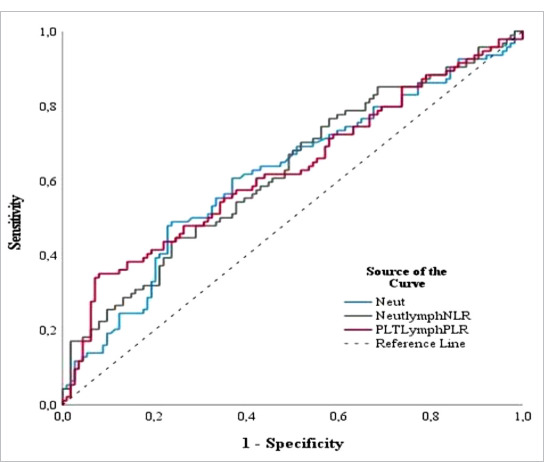
ROC curves of three markers for children with GI involvement

ROC curve analysis revealed that statistically significant predictors of GI involvement in IgAV children included NLR, PLR and neutrophil counts. The largest AUC (area under the curve) was demonstrated for the PLR (AUC = 0.627, p < 0.0001) (Fig. 3), which at the optimal cut-off value determined using the Youden index at the level of 132.97 showed a high specificity (90%) (Table 4).

**Table 4:** ROC curves analysis of NLR, neutrophils count and PLR for predicting GI involvement

Test Result Variable(s)	AUC	Sensitivity %	Specificity %	p value	Youden index
Neutrophil count	.616	48.9	76.3	.000	0.2525
NLR	.623	44.7	75.4	.000	0.2012
PLR	.627	35.1	76.3	.000	0.2721

Abbreviations: ROC, receiver operating characteristic; NLR, neutrophil-to-lymphocyte ratio; PLR, platelet-to-lymphocyte ratio; AUC, area under the curve.

## Discussion

Majority of our study findings such as incidence rate, male–female ratio, age of onset, IgA triggering factors and seasonal distribution confirmed the similar findings of other studies, which might indicate no peculiarities among different countries and centers [4, 11]

Our study covered only hospitalized patients, so easier cases didn’t fall in our scope. On the other hand, in Lithuania there was a high number of hospitalized patients with IgAV, even with mild disease forms with only skin involvement. This might be explained by the fact that Lithuania up to 2018 was an endemic zone for meningococcal B infection till vaccination for meningococcal B infection. The latter was included in national immunization plan after 2018. Mostly all patients with hemorrhagic rash, including IgAV patients, were hospitalized because of the possibility for the greater threat. After immunization started, inpatient IgAV patients decreased. It was another reason for the decrease in the number of IgAV inpatients during an observational period: COVID-19 pandemic led to dramatic changes in our daily life such as wearing masks, social distancing, quarantine. These measures seem to have resulted in decreased transmission of other infectious agents and it could be a reason why during pandemics an incidence of not only infectious diseases, but diseases triggered by infections, as IgAV, decreased [11, 13].

Although predictive factors for systemic involvement have been extensively studied, easily accessible and cost-effective laboratory parameters is still needed. There are several pro-inflammatory cytokines, such as tumor necrosis factor alfa (TNF-α), interleukin 1, interleukin 4, interleukin 6, and interleukin 8 may contribute to the pathogenesis of IgAV [5-6, 11, 14]. Yet these markers are costly and not available in many health centers.

Blood cell parameters, including white blood cell count and its subtype cell counts (neutrophils, lymphocytes and monocytes), and MPV are commonly used clinical indicators, with established detection methods and rapid results [1-2, 5-6, 12-14]. Blood cell count-derived inflammatory parameters, including NLR and PLR, are now recognized as biomarkers for treatment strategies in other rheumatic diseases [15-16] and novel biomarkers of chronic subclinical and inflammation in diabetes mellitus [17], cancer [18] and could be easily obtained from blood count.

Increased neutrophils are usually observed in cases of infection, as well as in cases of inflammation. IgAV is an inflammatory disease with predominant neutrophil response, and it is reasonable to assume that high levels of NLR are associated with the immune response in IgAV. Our findings, that the neutrophil, lymphocyte, platelet counts and NLR and PLR values were significantly higher in the IgAV group compared with control groups are similar to other studies [2, 4] and support this theory.

The association of NLR, PLR and MPV with the severe GI and kidney involvement in children with IgAV remains controversial [2, 5, 12-14], as our study results showed the same. As possible biomarkers of disease outcome, the appropriate values of these parameters are investigated to predict greater risk of GI or kidney involvement for children with IgAV.

IgAV with GI involvement showed that neutrophil and lymphocyte count, NLR and PLR levels are significantly higher in this group compared to those without GI involvement with no difference in MPV. Bowen Li analyzing 195 articles and 12 studies with totally 2168 patients involved found that higher NLR and lower MPV significantly correlated with the presence of the severe GI involvement in children with IgAV [5]. Our study data on NRL and even MPV could be used to predict GI disease form of IgAV and might require another treatment option.

Unfortunately, no laboratory predictors of IgAVN in children were found, except the older age at IgAV onset. This confirms the results found in other studies. Perhaps older patients should be monitored more carefully and for the longer period for kidney involvement performing urine tests regularly, which are easily available and cheap [1, 19]. Few studies showed significantly higher NLR in IgAVN group and values were higher than 2.62±1.95 in Yuang Y et al. study and 3.6±2.7 in Woo Kyung Kim et al. study, but PLR and MPV results were controversial [1-2, 4-5]. Perhaps, larger groups of patients are needed.

## Limitations

Our study has some limitations as only hospitalized patients were included and criteria for hospitalization differed according to epidemiological situation. Furthermore, IgAV patients were not on regular follow up intervals, which might have led to missing some outcome results.

## Conclusions

Easy obtained biomarkers, such as NLP, PLR could help to predict GI involvement in early disease stage, but are of no value for predicting kidney involvement. Older patients with male predominance have higher risk of kidney involvement. IgAV cases are reducing, proved to have low numbers of chronic kidney disease.

## List of Abbreviations

AUC – area under the curve

EULAR – European alliance of associations for rheumatology

GI – gastrointestinal

IgAV – Immunoglobulin A vasculitis

IgAVN – Immunoglobulin A vasculitis with nephritis

MPV – mean platelet volume

NLR – neutrophil-to-lymphocyte ratio

OR – Odds ratio

PLR – platelet-to-lymphocyte ratio

PRES – Paediatric rheumatology European society

PRINTO – Paediatric rheumatology international trials organization

ROC – receiver operating characteristic

SD – standard deviation

TNF-α – tumor necrosis factor alfa

URI – upper respiratory infection
